# The effect of nanoparticle size on the probability to cross the blood**-**brain barrier: an in-vitro endothelial cell model

**DOI:** 10.1186/s12951-015-0075-7

**Published:** 2015-03-04

**Authors:** Malka Shilo, Anat Sharon, Koby Baranes, Menachem Motiei, Jean-Paul M Lellouche, Rachela Popovtzer

**Affiliations:** Faculty of Engineering & the Institute of Nanotechnology and Advanced Materials, Bar-Ilan University, Ramat Gan, 52900 Israel; The Department of Chemistry & the Institute of Nanotechnology and Advanced Materials, Bar-Ilan University, Ramat Gan, 52900 Israel

**Keywords:** Blood**-**brain Barrier, Gold Nanoparticles, Barbiturate, bEnd.3 cells, Nanoparticle size

## Abstract

**Background:**

During the last decade nanoparticles have gained attention as promising drug delivery agents that can transport through the blood brain barrier. Recently, several studies have demonstrated that specifically targeted nanoparticles which carry a large payload of therapeutic agents can effectively enhance therapeutic agent delivery to the brain. However, it is difficult to draw definite design principles across these studies, owing to the differences in material, size, shape and targeting agents of the nanoparticles. Therefore, the main objective of this study is to develop general design principles that link the size of the nanoparticle with the probability to cross the blood brain barrier. Specifically, we investigate the effect of the nanoparticle size on the probability of barbiturate coated GNPs to cross the blood brain barrier by using bEnd.3 brain endothelial cells as an in vitro blood brain barrier model.

**Results:**

The results show that GNPs of size 70 nm are optimal for the maximum amount of gold within the brain cells, and that 20 nm GNPs are the optimal size for maximum free surface area.

**Conclusions:**

These findings can help understand the effect of particle size on the ability to cross the blood brain barrier through the endothelial cell model, and design nanoparticles for brain imaging/therapy contrast agents.

## Background

The diagnosis and therapy of central nervous system (CNS) and brain pathologies, including Parkinson’s disease, Alzheimer’s disease, epilepsy and glaucoma, are inadequate because of the limited ability to deliver drugs and imaging contrast agents across the blood brain barrier (BBB) [1]. The BBB separates circulating blood from the brain extracellular fluid (BECF) in the CNS and protects the brain from various circulating toxins and infected cells [2]. It is estimated that more than 98% of small molecular weight drugs and practically 100% of large molecular weight drugs developed for CNS pathologies do not readily cross the BBB [3]. Therefore, it is extremely important to investigate the BBB penetration mechanism in order to treat and prevent brain disorders. To improve brain penetration for therapeutic agents, medicinal chemistry and pharmaceutical technology-based strategies have been explored and developed widely [4].

During the last decade, nanoparticles have gained attention as promising drug delivery agents that can transport across the BBB and increase the uptake of appropriate drugs in the brain [1,4-6]. Nanoparticles increase the duration of drug circulation in the blood, which facilitates drug ability to interact with specific molecules expressed on the luminal side of BBB endothelial cells, and consequently to cross the BBB. In addition, nanoparticles can be engineered to provide designed functionalities using standard procedures in nanotechnology [7-10]. The type and number of linkers on the surface of the nanoparticles, as well as the size of the nanoparticles themselves, can be modulated to increase their ability to cross the BBB. Gold nanoparticles (GNPs) have attracted enormous scientific interest due to properties that make them useful for a large variety of biological and chemical applications [11-14]. Their major advantages are the ability to be synthesized at diverse sizes, their chemical stability and their unique optical properties [7,15,16]. Their surface has a strong binding affinity towards thiol, disulfide and amine groups, which allows by simple chemistry, surface conjugation with various peptides, proteins, antibodies and other biomolecules [17-19]. Most importantly, GNPs can be traced noninvasively in vivo by CT imaging, due to their high atomic number [20-24], and can be quantitatively detected ex vivo by atomic absorption methods.

It has been well demonstrated that the size, coating and surface charge of nanoparticles have a crucial impact on the intracellular uptake process [25]. Several studies, which investigated the effect of the size of nanoparticles on cellular uptake, revealed different conclusions; One study [25] compared between 14, 30, 50, 74 and 100 nm GNPs and reported that the highest uptake was detected for 50 nm GNPs, as reported in other studies [26,27], while another group showed that 20 nm GNP gave the best results [28]. However, differences in material, size and shape of the nanoparticles, variability between receptors (e.g., degree of receptor overexpression) and divergent cell types, make it difficult to draw definite design principles across these studies [25,29].

In the present study, the effect of nanoparticle size on the probability to cross the BBB was investigated using the bEnd.3 brain endothelial cell model. The BBB is formed from a single layer of endothelial cells, which are joined by tight junctions, in the cerebrovascular capillaries and end-feet astrocytes that cover the surface area of the capillary. Therefore, the bEnd.3 model is the first barrier between the blood and the brain and has a critical influence on the probability to cross the BBB. This BBB cellular model has been widely used in the last few years, and is considered an attractive candidate for an in vitro model of the BBB [30-32].

Normal transition through the BBB can be performed by diffusion transport, carrier systems and receptor-mediated endocytosis [1]. Entrance of GNPs to these cells can indicate penetration through the BBB. This in vitro model does not represent abnormal BBB such as in brain tumors [33].

GNPs of various sizes (20, 50, 70 and 110 nm) were synthesized and coated with barbiturate, which is a molecule that can easily penetrate the BBB [34]. Therefore, coating GNPs with barbiturate molecules will facilitate their penetration through the BBB, both for therapy and imaging applications. While several studies reported that receptor-mediated endocytosis has been shown to be the most efficient transport mechanism through the BBB, especially for large molecules, proteins and nanoparticles [18,25], we hypothesize that there is an interaction between the barbiturate molecule and the brain endothelium that triggers particle uptake (probably through pinocytosis [35]).

## Results and discussion

### GNP synthesis, conjugation and characterization

**Figure 1:**
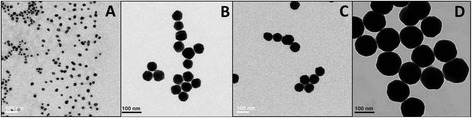
**TEM images of (A) 20, (B) 50, (C) 70 and (D) 110 nm GNPs (scale bar 100 nm).**

We have successfully synthesized GNPs in various sizes, ranging from 20 nm up to 110 nm. Particles size, shape and uniformity were measured using transmission electron microscopy (TEM) and proven to be 20, 50, 70 and 110 nm diameter spheres (Figure 1). It is demonstrated that the smallest GNPs (20 nm) have a relatively large size distribution (20%), while larger GNPs are more homogeneous, with have a very narrow size distribution (~2%). The average sizes that were obtained from the TEM were 18 ± 4 nm, 51 ± 1 nm, 67 ± 1 nm and 108 ± 1 nm.

**Figure 2:**
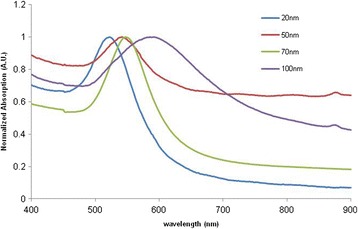
**UV–vis spectroscopy of 20, 50, 70 and 110 nm GNP.** Each size exhibits a peak at a different wavelength: 525, 530, 540 and 570, respectively. When the GNPs are enlarged, there is a red shift in the surface plasmon resonance (SPR) peak of the particles, from 525 nm to 570 nm.

The difference in GNP size was also obtained using UV–vis spectroscopy. Figure 2 shows a correlation between the size of the GNP and its spectrum. It can be seen that when the GNPs are enlarged, there is a red shift in the surface plasmon resonance (SPR) peak of the particles, from ~525 nm to ~580 nm [36].

**Figure 3:**
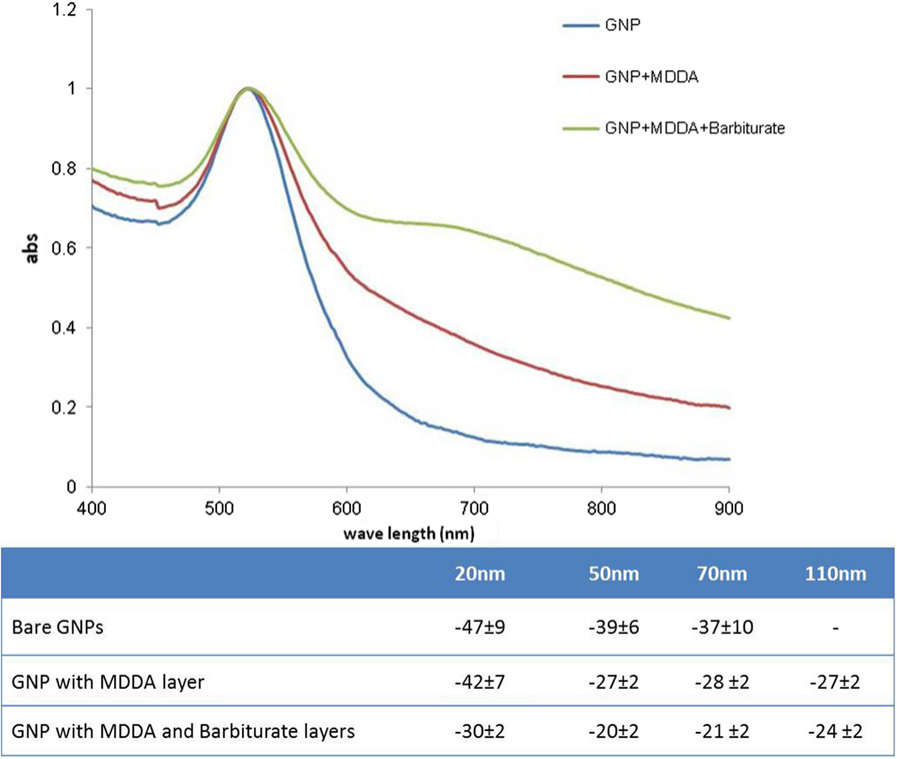
**Characterization of the GNPs.** Top: UV–vis Spectroscopy of bare GNPs, MDDA coated GNPs and MDDA + Barbiturate coated GNPs, for 20 nm GNPs. An expanded signal was observed following each layer of coating, confirming the chemical coating. Bottom: zeta-potential measurements at the various stages of GNP coatings for each GNP size. The significant difference obtained (both by zeta-potential and UV–vis Spectroscopy) following each chemical step demonstrates the efficiency of the chemical coating.

The size difference and efficiency of the GNP coating (both MDDA and barbiturate) were confirmed by the decrease (absolute value) of the zeta-potential and by the UV–vis Plasmon resonance shift and broadening (Figure 3). An expanded signal was observed following each layer-coating because the organic substance absorbs more energy from the irradiated light. The GF-GNPs were stable for up to three months, confirmed by retention of their plasmon resonance.

**Figure 4:**
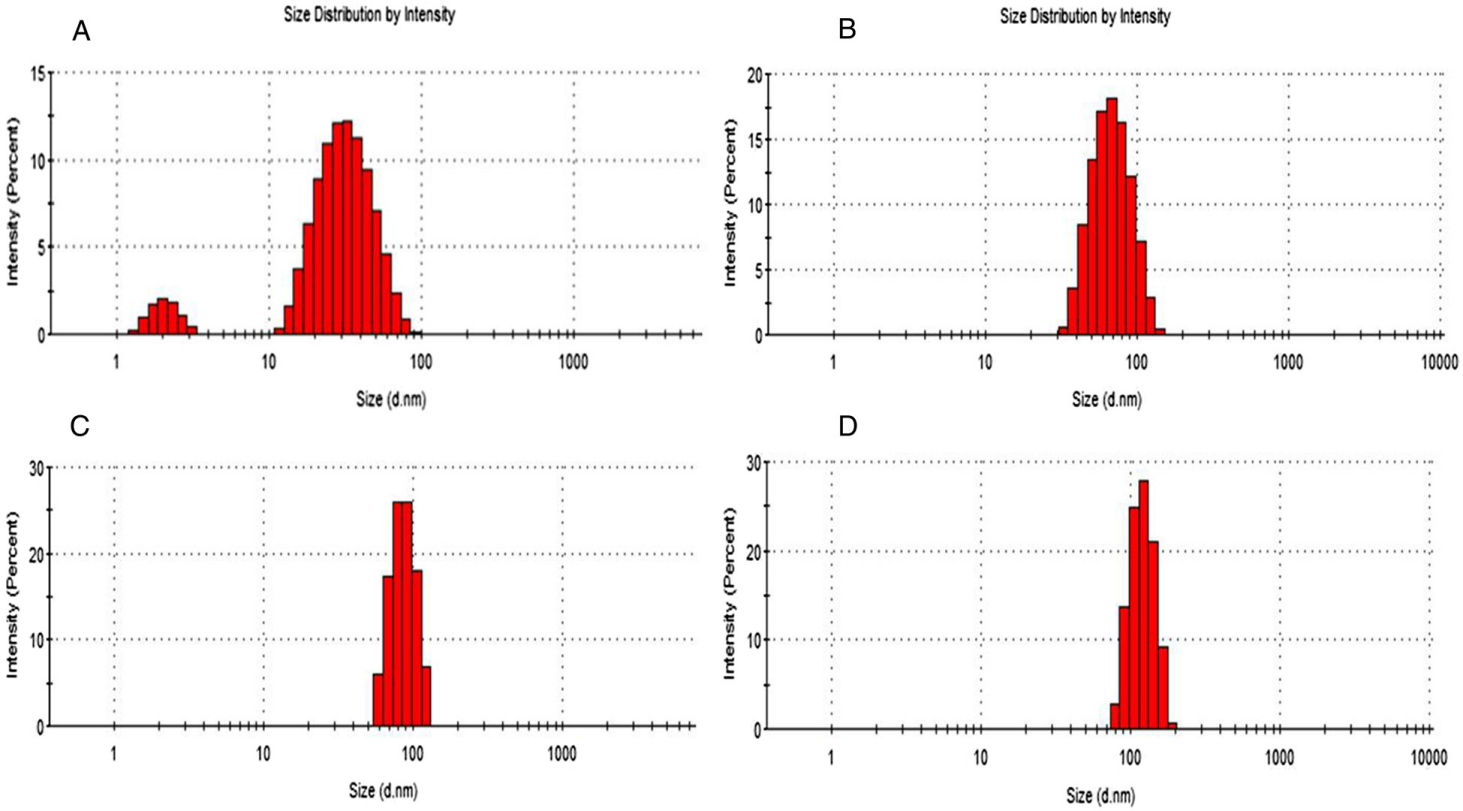
**DLS size distribution analysis for (A) 20, (B) 50, (C) 70 and (D) 110 nm GNPs.** The average sizes obtained from the DLS were 23 ± 13, 65 ± 22, 84 ± 26 and 120 ± 30 nm for the 20, 50, 70 and 110 nm GNPs, respectively.

Size distribution of the barbiturate coated GNPs was also determined using DLS (Figure 4). The average sizes obtained from the DLS were 23 ± 13, 65 ± 22, 84 ± 26 and 120 ± 30 nm for the 20, 50, 70 and 110 nm GNPs, respectively. The larger size of the particles obtained by the DLS is due to the hydrodynamic diameter of particles. Similar to the TEM results, the 20 nm GNPs have a relatively large size distribution, while the larger GNPs are found to be more homogeneous.

### In vitro cell experiments

In a control experiment, we compared between the intracellular uptake of barbiturate coated GNPs and mPEG coated nanoparticles, both of size 20 nm. Results showed that barbiturate coated GNPs had high uptake in the bEnd.3 model, while the control GNPs showed a negligible amount of uptake by the cells, which could not be detected by FAAS (Data not shown).

**Figure 5:**
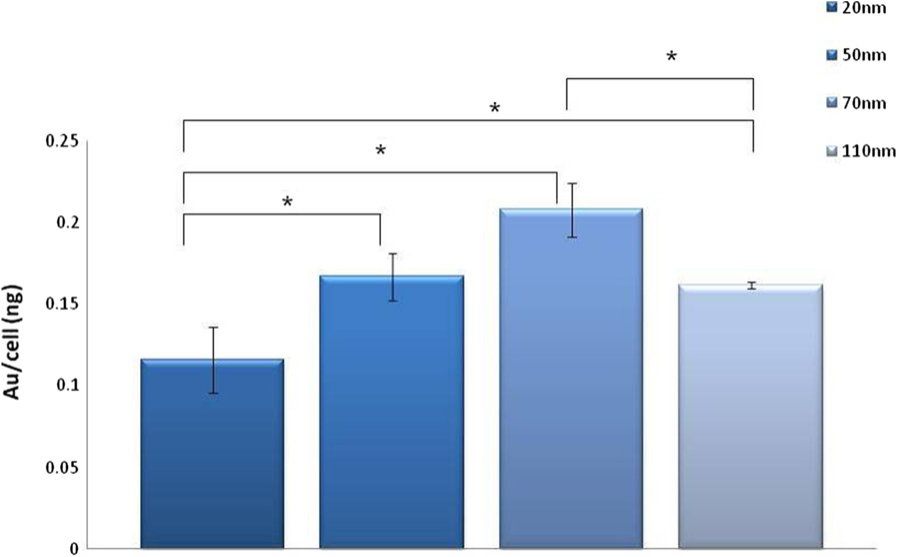
**Quantitative measurements using FAAS of Au per cell, for different sizes of GNPs.** Each cell sample contained 1.3×10^6^ cells. Results clearly demonstrate that 70 nm GNPs produce the largest amounts of gold uptake per cell. Results are presented as the total amount of gold (ng) per cell, mean ± S.D of three samples. *P < 0.05, Student’s t-test.

**Figure 6:**
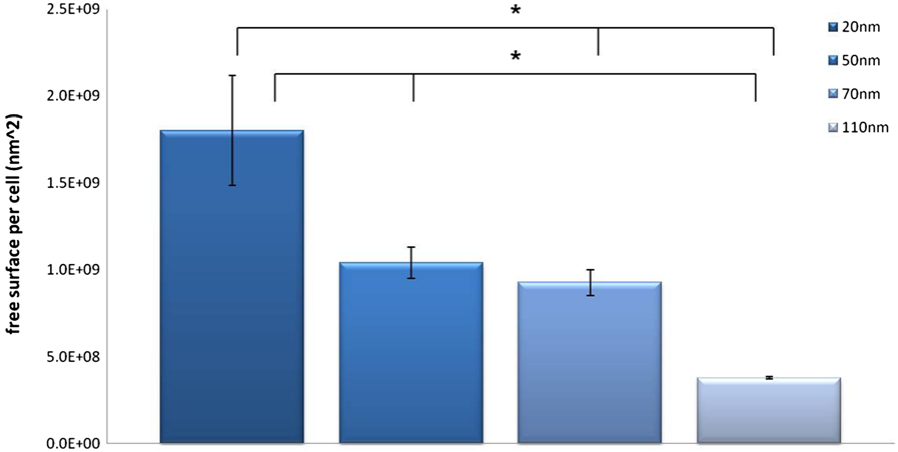
**Calculated total free surface area per cell, for different sizes of GNPs.** The free surface area decreased with the increase in GNP size. Results are presented as GNP surface area per cell, mean ± S.D of three samples. *P < 0.05, Student’s t-test.

The results showed that intracellular uptake of GNPs is clearly dependent on GNP size. Figure 5 shows the total amount of gold per cell for the various sizes of GNPs (20, 50, 70 and 110 nm). Results clearly demonstrate that 70 nm GNPs produce the largest amounts of gold uptake per cell (0.21 ± 0.03 ng, about 90% the GNPs), while for the 20 nm GNPs the uptake was only 0.12 ± 0.03 ng (about 50% of the GNPs) per brain endothelial cell. A T-test performed on these results showed a significant difference (P value <0.05) between the different sizes (Figure 5). Once we had quantitatively measured (using FAAS) the total amount of gold bound to a single cancer cell, the exact number of nanoparticles and the GNP surface area per cell could be calculated. When the total free surface area was examined, 20 nm GNPs had the maximum free surface area per cell. The free surface area decreased with the increase in GNP size (Figure 6). A T-test was performed on these results and showed a significant difference (P value <0.05) between the different sizes. Table 1 shows the total Au mass, the number of GNPs of different sizes and the surface area of the GNPs bound to a single brain endothelial cell.

**Table 1:** **Quantitative flame atomic absorption measurements of GNP total Au mass (ng), total GNP volume (nL) and total free surface area (μm** ^**2**^ **) per single cell**

**GNP size (nm)**	**Au/cell (ng)**	**Total volume (nl)**	**Total surface (μm** ^**2**^ **)**
20	0.12 ± 0.03	6E^−6^ ± 1.05^−6^	1800 ± 545
50	0.17 ± 0.02	8.7^−6^ ± 7.6^−7^	1040 ± 158
70	0.21 ± 0.03	1.08^−5^ ± 8.6^−7^	926 ± 126
110	0.16 ± 0.004	8.4^−6^ ± 1.04^−7^	379 ± 8

**Figure 7:**
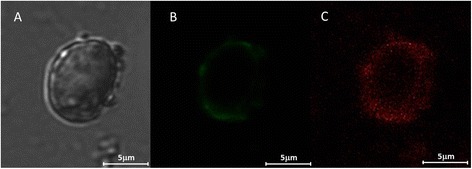
**Confocal images of bEnd.3 cells after 30 min of incubation with 70 nm fluorescent coated barbiturate-GNPs complex. A**: A bright field, **B**: Cell membrane staining and **C**: fluorescent coated barbiturate-GNPs. The pictures were taken at the midsection of the cell. Sections were imaged using Leica TCS SP5 with Acousto-Optical Beam Splitter microscope.

We have further investigated, using confocal microscopy and fluorescent coated barbiturate-GNPs, the interaction between the nanoparticles and the bEnd.3 cells. Serial *z*-sections of the cells, each 0.5 μm in thickness, demonstrated fluorescence activity in all the sections between 2 and 7 μm from the surface of the cells indicating that large part of the nanoparticles were internalized by the cells, while some nanoparticles also bound to the cell surface. Figure 7 shows confocal microscopy of midsection of bEnd.3 cell after incubation of 30 min with barbiturate coated GNPs.

Results demonstrated that intracellular uptake of GNPs is strongly dependent on GNP size. Different biomedical applications require different considerations: for example, when GNPs serve as CT contrast agents or as drug delivery carriers (by drug encapsulation into the GNPs), the highest amount of gold is required (70 nm). However, when GNPs serve as drug delivery carriers by binding the drug molecules to the surface of the GNPs, the highest free surface area is needed, and thus the appropriate size would be 20 nm.

## Conclusions

In order to develop general design principles for nanoparticles to be used as in vivo imaging contrast agents or drug delivery agents, this study investigated the effect of nanoparticle size on the probability to cross the BBB, using the endothelial brain cell model. Results showed that the intracellular uptake of GNPs is dependent on GNP size, and the appropriate size should be determined according to the desired application. These results may accelerate the development of general design principles for GNPs to cross the BBB, and help to meet the great challenge of providing treatments and imaging capabilities for brain diseases and disorders.

## Methods

### GNP-synthesis and conjugation

#### Synthesis of 20 nm GNPs

GNPs were synthesized by citrate reduction of Hydrogen tetrachloroaurate(III) trihydrate (HAuCl_4_, Strem) [37]. 414 μL of 50% w/V of HAuCl_4_ solution in 200 mL purified water was boiled in an oil bath on a heating plate while being stirred. After boiling, 4 mL of a 10% trisodium citrate solution was added and the mixture was stirred while boiling for another 5 minutes. The reducing agent, citrate, has a limited ability to synthesize GNPs that are larger than 30 nm, and therefore, the reducing agent MSA (2-mercaptosuccinic acid, Molekula), which enables synthesis of larger GNPs, was used. For GNP synthesis, 15 ± 1.5 nm gold seeds were first prepared. A mixture of MSA and HAuCl_4_ solutions leads to the growth of the gold seeds. Various amounts of MSA and gold solution were added for each GNP size, as detailed below.

For the control experiment, GNPs of size 20 nm were synthesized and coated with a layer of Methoxy-PEG-SH (mPEG-SH). This layer reduces nonspecific interactions and increases blood circulation time of the nanoparticles [38,39].

#### Synthesis of seed solution for GNPs at 50, 70, and 110 nm

10 mL of purified water was mixed with 10.4 μL of 50% w/V of HAuCl_4_ solution. The solution was stirred and boiled on a heating plate. To the stirred solution, 100 μL of Na_3_ citrate (8.8% weight percentage) was added and the mixture was stirred and boiled for another 5 minutes (the solution was diluted with water to a volume of 50 mL).

#### Synthesis of 50, 70 and 110 nm GNPs in Growth solution

For synthesis of 50 nm GNPs, 200 mL of purified water was mixed with 6.5 mL seed solution. 88 μL of 50% w/V of HAuCl4 and 7.5 mL 0.04 M MSA solution was added while stirring, and the mixture was stirred for another half hour. For synthesis of 70 nm GNPs, 176.8 μL of 50% w/V of HAuCl_4_ solution and 15 mL 0.04 M MSA solution was added while stirring. The mixture was stirred for another half hour. For synthesis of 110 nm GNPs, another 3 portions of 176.8 μL of 50% w/V of HAuCl_4_ solution and 15 mL 0.04 M MSA solution was added after the half hour of stirring.

### Conjugation of MDDA and Barbiturate to the GNPs

**Figure 8:**
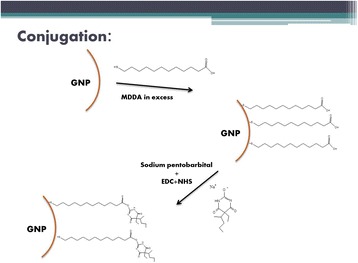
**Synthesis of GNPs.** Schematic diagram of the synthesis of GNPs and functionalization with barbiturate. In order to conjugate the glucosamine to the GNP, the linker 12-Mercaptododecanoic acid (MDDA) was utilized. EDC and NHS were added in order to activate the carboxylic acids of the linker. One side of the MDDA chain (thiol) connects to the gold via semi-covalent bonding, while the other side, carboxylic acid, binds to the negatively charged oxygen of the barbiturate.

The linker MDDA (12-Mercaptododecanoic acid, Sigma-Aldrich) conjugates between the GNP and the barbiturate. One side of the MDDA chain (thiol) connects to the gold via semi-covalent bonding, while the other side of the MDDA chain, carboxylic acid, binds to the negatively charged oxygen of the barbiturate. To each solution of the various GNP sizes, MDDA was added in excessive amounts, and the mixture was stirred for another four hours. Following this step, the solutions were centrifuged in order to reach higher concentrations. Next, the activating agents EDC (1-Ethyl-3-(3-dimethylaminopropyl) carbodiimide HCl, Thermo Scientific) and NHS (N-Hydroxysulfosuccinimide sodium salt, Chem-Impex International) were added to the mixture together with the barbiturate, and stirred overnight (Figure 8). The solutions were centrifuged again in order to increase concentration.

#### GNP characterization

The size, shape and uniformity of the GNPs were measured using transmission electron microscopy (TEM, JEM-1400, JEOL). In addition, GNP size was measured using ultraviolet–visible spectroscopy (UV-1650 PC; Shimadzu Corporation, Kyoto Japan) and Dynamic Light Scattering (DLS). Conjugation of the barbiturate to the linkers was verified using Zeta potential and Fourier Transform Infrared (FTIR).

### Brain endothelial cell model

As an in vitro model of the BBB, the mouse brain endothelial cell line (bEnd.3, American Type Culture Collection Manassas, VA) was used [30,31,40]. Cells were grown in DMEM with 4.5 g/L glucose, 10% FBS, 3.7 g/L sodium bicarbonate, and 4 mM glutamine, and maintained in a humidified cell culture incubator at 37°C.

### In vitro experiments

Each different sized group of GNPs was incubated with the brain endothelial cells (1.3 × 10^6^) for a quantitative cell uptake study (each experimental group was run in triplicate). The procedure was identical for all groups: excessive amounts of GNPs were added to total medium volume of 5 ml (identical amounts of gold per cell) and the incubation time was 30 minutes at 37°C. The four cell groups were incubated with 10 μL of either 20, 50, 70 or 110 nm GNPs (30 mg/mL). After incubation, the medium was washed twice with PBS, followed by trypsin treatment. The cells were centrifuged twice (7 minutes at 1000 rpm) in order to get rid of the unbound nanoparticles. Finally, aqua-regia was added to the cells for ICP-MS gold detection. The total amount of gold was compared between the various GNP sizes.

### Quantitative measurements of GNP uptake by brain endothelial cells

Flame Atomic Absorption Spectroscopy (FAAS, SpectrAA 140, Agilent Technologies) was used in order to determine the amount of the different sized GNPs within the cells. Samples were melted with aqua regia acid (a mixture of nitric acid and hydrochloric acid in a volume ratio of (1:3)), filtered and diluted to a final volume of 5 mL. Gold concentration was determined according to absorbance values, compared to a calibration curve that was prepared with known gold concentrations. All samples were analyzed by FAAS under the same experimental conditions.

### Synthesis of fluorescent coated barbiturate-GNPs

Rodamine B and PEG-amine were attached to the barbiturate-GNPs by stirring with EDC and NHS for one hour. The mixture was stirred for 3 hours and then centrifuged in order to remove excess rodamone B.

### Confocal microscopy experiment

Fluorescent coated barbiturate-GNPs were incubated with the brain endothelial cells for 30 min at 37°C. Then, cells were incubated with the fluorescent lipophilic dye, DiO (Biotium, Inc., Hayward, CA) diluted in DMSO in a final concentration of 10uM in the culture medium for 15 min at 37°C. The cells were subsequently washed three times in PBS prior to confocal imaging using Leica TCS SP5 with Acousto-Optical Beam Splitter microscope to acquire fluorescent and bright field images.

## Abbreviations

BBB: Blood brain barrier
CNS: Central nervous system
BECF: Brain extracellular fluid
GNPs: Gold nanoparticles
TEM: Transmission electron microscopy
SPR: Surface plasmon resonance
DLS: Dynamic Light Scattering
FTIR: Fourier Transform Infrared
FAAS: Flame Atomic Absorption Spectroscopy
